# Supplementary material for: Chromosome organization by one-sided and two-sided loop extrusion
Source: eLife. 2020 Apr 6;9:e53558. doi: 10.7554/eLife.53558 (PMC7295573; doi:10.7554/eLife.53558)
Supplement: Transparent reporting form [file elife-53558-transrepform.docx]

***eLife’s* transparent reporting form**

We encourage authors to provide detailed information *within their submission* to facilitate the interpretation and replication of experiments. Authors can upload supporting documentation to indicate the use of appropriate reporting guidelines for health-related research (see [EQUATOR Network](http://www.equator-network.org/%20)), life science research (see the [BioSharing Information Resource](https://biosharing.org/)), or the [ARRIVE guidelines](http://www.plosbiology.org/article/info:doi/10.1371/journal.pbio.1000412) for reporting work involving animal research. Where applicable, authors should refer to any relevant reporting standards documents in this form.

If you have any questions, please consult our Journal Policies and/or contact us: [editorial@elifesciences.org](mailto:editorial@elifesciences.org).

**Sample-size estimation**

- You should state whether an appropriate sample size was computed when the study was being designed
- You should state the statistical method of sample size computation and any required assumptions
- If no explicit power analysis was used, you should describe how you decided what sample (replicate) size (number) to use

Please outline where this information can be found within the submission (e.g., sections or figure legends), or explain why this information doesn’t apply to your submission:

Sample sizes for all simulation results can be found in the Supporting File 1, Supplementary Figure captions and are described in the Methods sections. We chose sample sizes such that the standard deviation of the mean of each data point were either less than 15% of the absolute value of the observable, or we display simulation results where the system has reached a steady-state as assessed by mean-square-displacement analyses, loop size statistics, and/or contact probability scaling.

**Replicates**

- You should report how often each experiment was performed
- You should include a definition of biological versus technical replication
- The data obtained should be provided and sufficient information should be provided to indicate the number of independent biological and/or technical replicates
- If you encountered any outliers, you should describe how these were handled
- Criteria for exclusion/inclusion of data should be clearly stated
- High-throughput sequence data should be uploaded before submission, with a private link for reviewers provided (these are available from both GEO and ArrayExpress)

Please outline where this information can be found within the submission (e.g., sections or figure legends), or explain why this information doesn’t apply to your submission:

All results reported in this paper were generated by simulations. As described above, simulations were performed with as many replicates (and/or temporal samples) such that the system has reached a steady-state conformation, and the relative standard errors for each data point were sufficiently small (<15% of the mean value). In Figure 3, we show Hi-C data from another publication with GEO accession number GSE96107. In Figure 4, we show Hi-C data from another publication with GEO accession number GSE68418.

**Statistical reporting**

- Statistical analysis methods should be described and justified
- Raw data should be presented in figures whenever informative to do so (typically when N per group is less than 10)
- For each experiment, you should identify the statistical tests used, exact values of N, definitions of center, methods of multiple test correction, and dispersion and precision measures (e.g., mean, median, SD, SEM, confidence intervals; and, for the major substantive results, a measure of effect size (e.g., Pearson's r, Cohen's d)
- Report exact p-values wherever possible alongside the summary statistics and 95% confidence intervals. These should be reported for all key questions and not only when the p-value is less than 0.05.

Please outline where this information can be found within the submission (e.g., sections or figure legends), or explain why this information doesn’t apply to your submission:

Standard errors and mean values are reported in figures as error bars on data points; in cases where the standard error was not displayed for visual clarity (Figure 2), it is stated that the standard error is smaller than 15% of mean, or smaller than the displayed data point.

(For large datasets, or papers with a very large number of statistical tests, you may upload a single table file with tests, Ns, etc., with reference to sections in the manuscript.)

**Group allocation**

- Indicate how samples were allocated into experimental groups (in the case of clinical studies, please specify allocation to treatment method); if randomization was used, please also state if restricted randomization was applied
- Indicate if masking was used during group allocation, data collection and/or data analysis

Please outline where this information can be found within the submission (e.g., sections or figure legends), or explain why this information doesn’t apply to your submission:

A sweep of simulation parameters was used initially to constrain parameters similar to “control” or “wild-type” data (see, e.g. Fudenberg et al., 2016). All modifications to the underlying assumptions and/or parameters (outside of the “control” values) were classified as the test sets. No randomization was performed.

**Additional data files (“source data”)**

- We encourage you to upload relevant additional data files, such as numerical data that are represented as a graph in a figure, or as a summary table
- Where provided, these should be in the most useful format, and they can be uploaded as “Source data” files linked to a main figure or table
- Include model definition files including the full list of parameters used
- Include code used for data analysis (e.g., R, MatLab)
- Avoid stating that data files are “available upon request”

Please indicate the figures or tables for which source data files have been provided:

Relevant source codes used in this publication have been deposited in:

https://github.com/mirnylab/one_sided_extrusion
